# β-Tricalcium Phosphate-Loaded Chitosan-Based Thermosensitive Hydrogel for Periodontal Regeneration

**DOI:** 10.3390/polym15204146

**Published:** 2023-10-19

**Authors:** Naiwen Tan, Maja Sabalic-Schoener, Linh Nguyen, Francesco D’Aiuto

**Affiliations:** 1 Periodontology Unit, UCL Eastman Dental Institute, 21 University Street, London WC1E 6DE, UK; naiwen.tan.20@ucl.ac.uk (N.T.); maja.1.sabalic@kcl.ac.uk (M.S.-S.); 2 Biomaterials and Tissue Engineering, UCL Eastman Dental Institute, Royal Free Campus, Rowland Hill Street, London NW3 2PF, UK; l.nguyen@ucl.ac.uk

**Keywords:** biocompatible materials, injectable hydrogel, chitosan, periodontitis, regenerative medicine tissue engineering, dental tissue repair

## Abstract

The current treatment for periodontitis is aimed at resolving gingival inflammation, whilst complete periodontal tissue regeneration is not predictable, and it represents a therapeutic challenge. Injectable biomaterials hold tremendous potential in dental tissue regeneration. This study aimed to investigate the ability of an injectable thermosensitive β-tricalcium phosphate (β-TCP) and chitosan-based hydrogel to carry cells and promote periodontal tissue regeneration. In this study, different concentrations of β-TCP-loaded chitosan hydrogels were prepared (0%, 2%, 4%, or 6% β-TCP, 10% β-glycerol phosphate, and 1.5% chitosan). The characteristics of the hydrogels were tested using rheology, a scanning electron microscope (SEM), X-ray diffraction (XRD), Fourier-transform infrared spectroscopy (FTIR), differential scanning calorimetry (DSC), degradation, and biological analyses. The new biomaterial showed a sol–gel transformation ability at body temperature and exhibited excellent chemical and physical characteristics, whilst the existence of β-TCP enhanced the structure and the properties of the hydrogels. The SEM confirmed the three-dimensional networks of the hydrogels, and the typical rheological properties of strong gel were observed. The EDX and XRD validated the successful incorporation of β-TCP, and similar patterns between different groups were found in terms of the FTIR spectra. The stable structure of the hydrogels under 100 °C was confirmed via DSC. Biological tests such as Alamar Blue assay and Live/Dead staining confirmed the remarkable biocompatibility of the hydrogels with pre-osteoblast MC3T3-E1 and human gingival fibroblast (HGF) cells for 14 days, and the results were validated with confocal imaging. This preliminary study shows great promise for the application of the β-TCP-loaded thermosensitive chitosan hydrogels as a scaffold in periodontal bone and soft tissue repair.

## 1. Introduction

Periodontitis is an inflammatory disease affecting the gingival tissues supporting the tooth, coupled with progressive tissue attachment and bone loss [1]. The classification of periodontitis was internationally accepted in 1999 [2], and periodontitis was further subdivided into four stages (defined by the severity, complexity, extent, and distribution of the disease) and three grades (defined by the rapid progression, anticipated treatment response, and effects on systemic health) at 2017 [3]. Periodontal regenerative therapy is aimed at recovering the lost periodontal soft and hard tissues. The ideal outcome of this treatment is to recreate the structure and the function of periodontal tissues, including new cement formation on the root surface, and new alveolar bone and periodontal ligament fibres connecting between cement and alveolar bone [4,5]. Successful regenerative therapy requires the appropriate coordination of three elements: the implanted cells that can create new tissue, a biomaterial acting as a scaffold or matrix to hold the cells, and biological signalling molecules that can direct the cells to form the desired tissue [6,7].

The current regenerative treatments are based on the concept of guided tissue regeneration (GTR) and bone grafting [8], which have also been combined. Although both regenerative techniques are widely applied in everyday clinical practice and have already shown some positive clinical outcomes, there are still challenges to achieving predictable regenerative outcomes due to the complex structure of the periodontium as well as the treated defect, and due to clinical case selection and technical challenges.

Hydrogels show great promise as a scaffold in tissue engineering because of their excellent physical, chemical, and biological properties. Due to their good biocompatibility and biodegradability, natural scaffolds have advantages for living tissue and cells [9]. Under certain stimulations, including physical, chemical, or biological stimuli, hydrogels can undergo sol–gel or gel–sol transitions. As applicable carriers, hydrogels can provide various functions by delivering different bioactive molecules. Nanoparticles [10], drugs [11], and cells [12] can be delivered to the targeted site to eliminate inflammation, enhance bone growth, or prevent the progress of the disease on-site. Specially designed hydrogels including postbiotic gel and ozone gel have already shown benefits to patients with periodontal diseases in clinical studies [13,14]. Chitosan-based hydrogels, a representative of natural hydrogels, elicit minimal immune responses and have antimicrobial ability as well as biocompatibility and biodegradability [15,16]. Injectable hydrogels represent a novel and non-invasive biomaterial which could be used to fill irregularly shaped defects [17]. When applied as a scaffold to reconstruct bone and soft tissue, chitosan-based hydrogel, however, still has some limitations. Indeed, they lack strong mechanical properties, and hence they need to be combined with other functional materials, including nanoparticles, polymers, and ceramics, to promote osteogenic differentiation and tissue regeneration [18].

As an inorganic ceramic material, β-tricalcium phosphate (β-TCP) is remarkable for its biocompatibility, high bioactivity, osteoconductivity, and thermodynamic stability. It can also increase the adhesion, proliferation, and differentiation ability of osteoblasts, which can simulate the mineralogical and structural bone composition [19]. It can directly bond to natural bone, and thanks to its biodegradability, it can be gradually absorbed and replaced by the new tissue. Β-TCP has already been approved and widely used in daily clinical practice [20]. The combination of β-TCP with organic materials could improve their physicochemical and biological characteristics.

Regenerative periodontal surgeries are routinely performed using a combination of biomaterials in the form of particles, membranes, and gels, and their application is technically sensitive. However, the new in situ gelling hydrogel scaffold combines a hydrogel that adapts well to the treated defect and beta-tricalcium phosphate particles to enhance osteogenic differentiation. The data to date suggest that it is biocompatible and, therefore, it could be used with cells such as endogenous granulation tissue cells. It is developed specifically with periodontal regenerative surgery in mind, aiming to improve handling and simplify application to increase the predictability of these procedures. Relevantly, the material could be used in patients who object to the use of porcine and bovine materials, marking a significant step forward in making regenerative periodontal surgery more inclusive and accessible.

This study aimed to investigate the ability of an injectable β-TCP-loaded chitosan-based thermosensitive hydrogel to act as an effective substrate and carrier of cells in periodontal regenerative therapy.

## 2. Materials and Methods

### 2.1. Hydrogel Preparation

**Figure 1:**
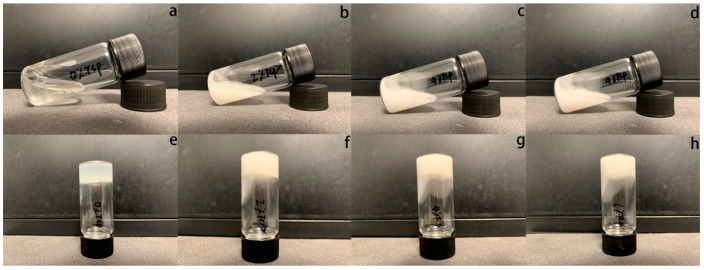
The gelification process of β-TCP-loaded chitosan hydrogel samples; 0%, 2%, 4%, and 6% β-TCP-loaded chitosan hydrogels were observed under room temperature (**a**–**d**) and at 37 °C (**e**–**h**) separately.

Three different concentrations of β-TCP (2%, 4%, and 6%, *w*/*v*%)-loaded chitosan hydrogels were prepared and compared to chitosan hydrogel alone (without β-TCP). Specifically, 300 mg of chitosan (448869, Sigma-Aldrich (St. Louis, MO, USA), molecular weight: 50,000–190,000 Da, deacetylation: 83%) was added into 10 mL of 1% lactic acid (252476, Sigma-Aldrich) and stirred for one hour. The well-mixed chitosan solution was kept in a 4 °C fridge overnight. β-Glycerophosphate (β-GP, G9422, Sigma-Aldrich) solutions were prepared by dissolving 2 g into 10 mL of deionized water and were kept in a 4 °C fridge overnight. Different amounts of β-TCP powder (0 mg, 400 mg, 800 mg, 1200 mg) (49963, Sigma-Aldrich) were added into the chitosan solution under magnetic stirring in an ice bath separately, and then the β-GP solution was added into the chitosan solution drop by drop under the same condition. At last, 1.5% chitosan (*w*/*v*%), 10% β-GP (*w*/*v*%) hydrogels with different β-TCP concentrations (0%, 2%, 4%, 6%, *w*/*v*%) were prepared and put into a 37 °C water bath to observe the sol–gel transition behaviour through the inverted test tube technique [21,22] as shown in Figure 1. All the samples were stored at 4 °C for the next experimental steps.

### 2.2. Morphology of the Samples Observed through Scanning Electron Microscope (SEM) and Energy Dispersive X-ray (EDX) Analysis

The hydrogel samples were kept at 37 °C overnight to allow for complete gelification for the next step of testing. These gelled hydrogels were then freeze-dried to remove water components. After being coated with palladium (Polaron E5000 Sputter Coater, Quorum Technologies, Laughton, UK), the morphology of the hydrogel was observed via scanning electron microscopy (Zeiss Sigma, Oberkochen, Germany) (EHT = 10 kV, WD = 7.8 mm). EDX analysis was also performed.

### 2.3. X-ray Diffraction (XRD) Analysis

The freeze-dried hydrogel samples were ground into powder and an XRD analysis of the powder samples was performed with a Bruker D8 Advance Diffractometer (Karlsruhe, Germany) to determine the phase composition. The scan range was set to 2θ from 10 to 100 degrees.

### 2.4. Spectroscopic Analysis

The Fourier-transform infrared (FTIR) spectra of the freeze-dried powder samples were analysed using an FTIR spectrometer (ATR-FTIR, System 2000, PerkinElmer, Seer Green, UK) at a resolution of 4 cm^−1^ to identify the functional groups of the β-TCP-loaded chitosan hydrogel. The spectra were recorded from 400 to 4000 cm^−1^.

### 2.5. Thermal Analysis

Differential scanning calorimetry (DSC25, TA Instruments, New Castle, NSW, USA) was used to analyse the heat capacity of the hydrogel samples. The weighed samples were put into Tzero^®®^ Pans and sealed with Tzero^®®^ lids. Together with an empty pan set as a reference, the pans with samples were heated from 0 °C to 250 °C at a ramp rate of 10 °C/min. The data were reported using TRIOS software (version 5.1.1.46572).

### 2.6. Rheological Analysis

The rheological characterization of these hydrogels was tested with a rotational rheometer (HAAKE Viscotester iQ Rheometers, Thermo Scientific, Walthman, MA, USA). The lineal visco-elastic range of the hydrogels was tested first to determine an appropriate strain value. Next, the viscoelastic moduli G′ and G″ were evaluated as functions of temperature, time, and frequency separately. Each group had three replicate samples.

### 2.7. Degradation Analysis

The degradability of the hydrogels was carried out in PBS, 10 μg/mL of lysozyme (L1667, Sigma-Aldrich), and 100 μg/mL of lysozyme and artificial saliva (AS) (SAE0149, Sigma-Aldrich) at 37 °C, respectively. One millilitre of the different hydrogels was put into Sterilin^TM^ 7 mL Plystyrene Bijou Containers (129B, Thermo Scientific^TM^) separately and left in a 37 °C incubator overnight for gelation, followed by adding 1 ml of the different medium into each bottle. The medium was replaced every day in the first week and every other day in the following three weeks. Each group had four replicates. The initial weights were measured with a weighing scale (Avery Weigh-Tronix PA224, OHAUS^®^, Nänikon, Switzerland) and the wet weights of the samples were measured on days 1, 2, 3, 7, 14, 21, and 28. The degradation process was reflected by measuring the weight remaining, and the weight remaining ratio (WRR) was calculated using the following formula:

WRR (%) = W_t_/W_0_ × 100

W_0_ and W_t_ are the weights of the hydrogels at baseline and after degradation, separately.

### 2.8. Biological Analyses

Cell culture. Osteoblastic cell line MC3T3-E1 (99072810, Sigma-Aldrich) and human gingival fibroblasts (HGF, CRL-2014^TM^, ATCC, Manassas, VA, USA) were cultured in an MEM-based growth medium (11095080, Gibco, Billings, MT, USA) with 10% foetal bovine serum (F7524, Sigma-Aldrich) and 1% penicillin–streptomycin (15140122, Gibco), and a DMEM-based growth medium (D6429, Sigma-Aldrich) with 10% foetal bovine serum and 1% penicillin–streptomycin, separately, in a humidified incubator at 37 °C in 5% CO_2_. The MC3TC-E1 and HGF (passage 6) were ready for cell viability test until cells attained >90% confluency.

Cell viability and proliferation studies using Alamar Blue assay. The hydrogel samples were sterilised before being mixed with a cell suspension. Specifically, the chitosan solution was sterilised for 60 min at 110 °C in an autoclave, the β-GP solution was sterilised via a 0.2 μm pore size syringe filter, and the β-TCP powder was exposed to UV light for 30 min before being mixed with the chitosan solution.

One hundred microliters of the prepared hydrogel solution samples were mixed with the cell suspension of both types of cells (1.5 × 10^5^ cells/well) and added into separate wells of 24-well tissue culture plates, and then the plates were incubated at 37 °C for 30 min to form the hydrogels, followed by adding 1 mL of the complete culturing medium. The medium was replaced every other day.

The Alamar Blue Cell Viability Reagent (DAL1025, Thermo Fisher Scientific, Waltham, MA, USA) was used to determine the cell viability and proliferation. Specifically, 400 μL of a 10% (*v*/*v*) reagent dissolved in a complete medium was added to each well after the culture medium was removed. The plate was incubated for 4 h at 37 °C in the dark, followed by a fluorescence intensity measurement using a Biotek FLx800 microplate reader (Winooski, VT, USA) (excitation: 540/35 nm, emission: 600/40 nm). Each group had three replicate samples.

Cell viability study using Live/Dead staining. The cell viability on chitosan hydrogel samples was further tested with a Live/Dead^TM^ Viability/Cytotoxicity Kit (L3224, Thermo Fisher Scientific) using HGF cells at days 1, 3, 7, and 14. Similarly, one hundred microliters of the sterilised samples were mixed with the HGF cell suspension (1.5 × 10^5^ cells/well) and added into separate dishes (Nunc^TM^ Glass Bottom Dishes, 150680, Thermo Fisher Scientific), followed by transferring the dishes into an incubator at 37 °C for 30 min. Two millilitres of the complete medium was added to the plate after the gel formation was achieved and the medium was replaced every 2 days. To perform the viability assay, 5 μL of calcein AM (Component A) and 20 μL of ethidium homodimer-1 (Component B) were added to 10 mL of PBS to create a staining solution. The medium was removed from the dishes and then 2 mL of the staining solution was added to the dish, followed by incubation for 30 min at room temperature in the dark. Finally, the images of the stained live and dead cells were collected using a confocal microscope (Aurox, Abingdon, UK) and Visionary software (version 2, Aurox, UK).

### 2.9. Statistical Analysis

The statistical analyses were conducted using a one-way ANOVA with a Tukey post-test analysis, and the data are presented as a mean with a standard deviation. The difference was considered significant if *p* < 0.01.

## 3. Results

### 3.1. Scanning Electron Microscope (SEM) and Energy Dispersive X-ray (EDX) Analysis

**Figure 2:**
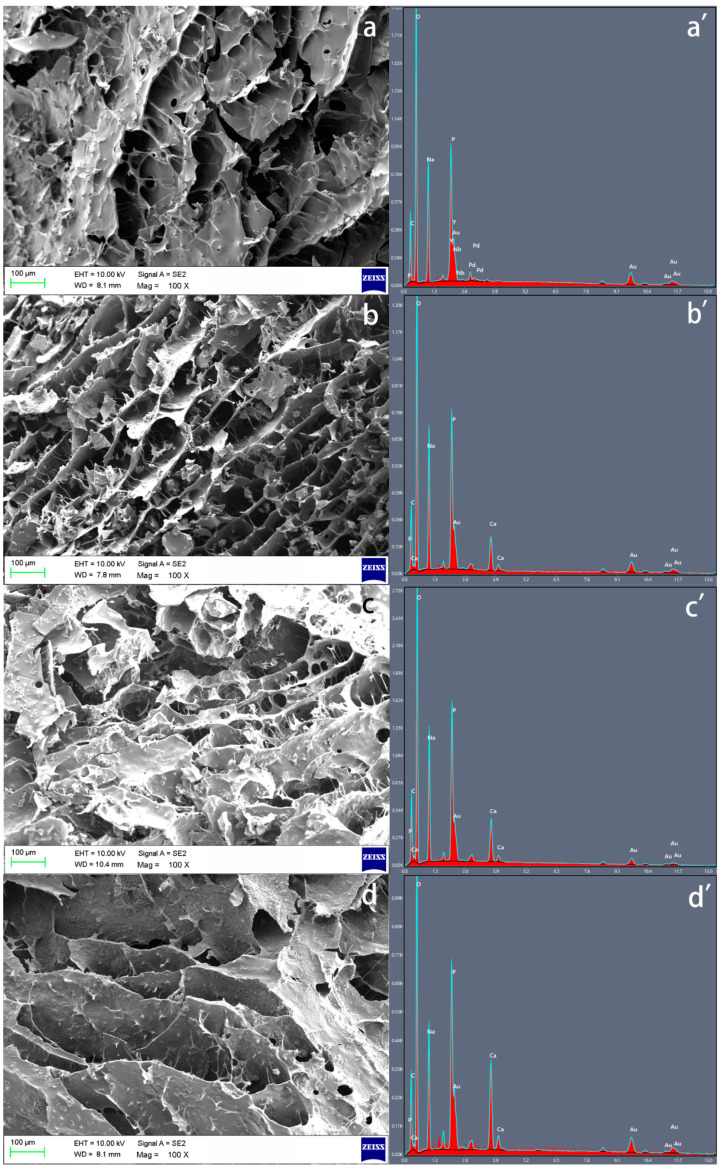
The different structures of β-TCP-loaded chitosan samples; 0% β-TCP (**a**), 2% β-TCP (**b**), 4% β-TCP (**c**), and 6% β-TCP (**d**) can be observed in SEM images. The EDX spectra of 0% β-TCP (**a′**), 2% β-TCP (**b′**), 4% β-TCP (**c′**), and 6% β-TCP (**d′**) were examined separately.

The SEM results of three freeze-dried hydrogel samples confirmed their porous structure, whilst the increase in the β-TCP concentration did not affect the porosity of the hydrogel but could strengthen the fibre content (Figure 2a–d). The presence and the differences of calcium and phosphate from β-TCP in the hydrogels were confirmed based on the EDX spectra (Figure 2a′–d′).

### 3.2. X-ray Diffraction (XRD) Analysis

**Figure 3:**
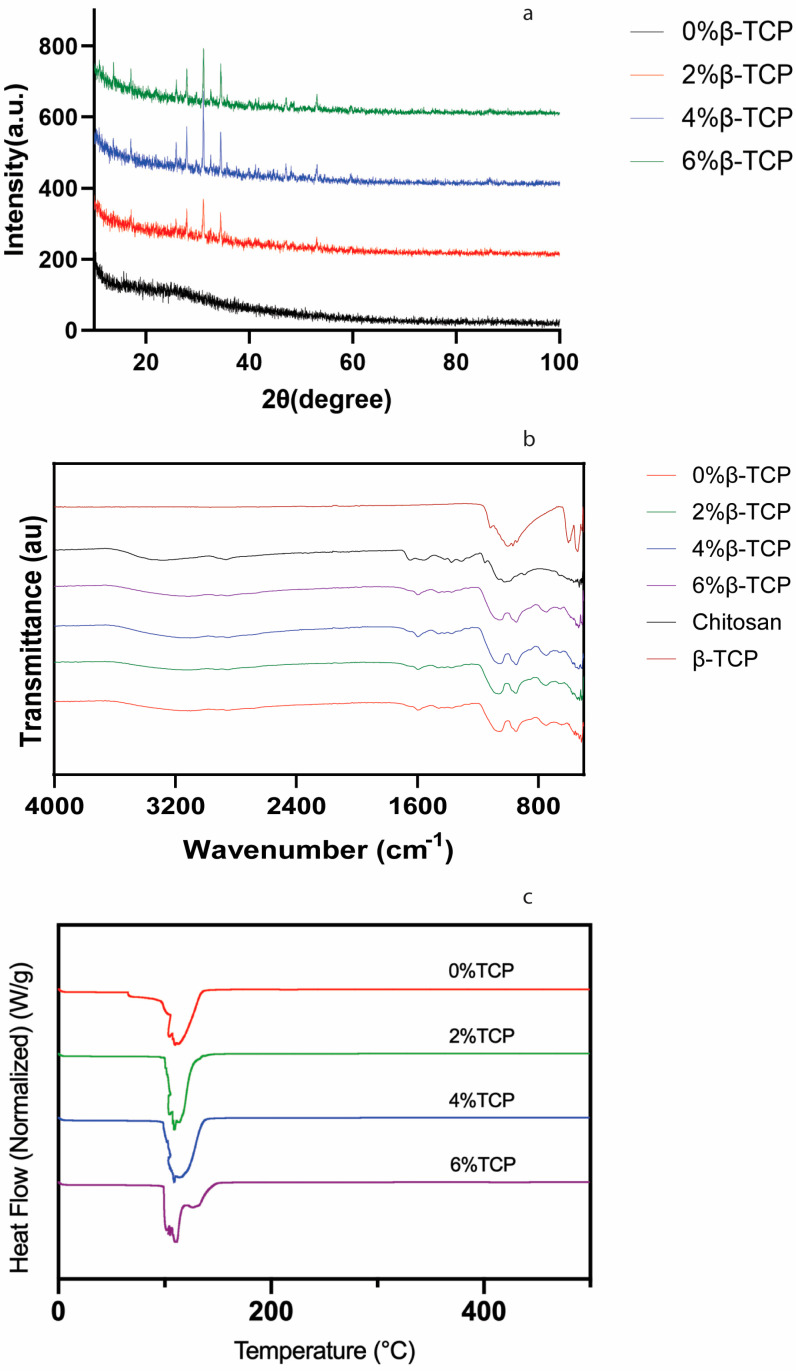
The X-ray diffraction characteristics, FTIR spectra of freeze-dried samples, and DSC of fresh samples are shown in (**a**–**c**) separately.

A marked peak at 32 degrees in the hydrogel samples containing β-TCP was observed, but this peak could not be found in the control group without β-TCP, indicating the presence of β-TCP (Figure 3a). The remarkable peak at 2θ = 12° and 20° corresponds to chitosan, and it has been reported previously [23].

### 3.3. Spectroscopic Analysis

Four hydrogel samples showed similar spectrum characteristics (Figure 3b). The peak at 1067 cm^−1^ can be found in four hydrogel samples, which can be assigned to –C–O–C– in the glycosidic linkage of chitosan [24]. The spectrum characteristics of the chitosan hydrogels with β-TCP and without β-TCP were very similar, likely because β-TCP was only physically mixed with the chitosan hydrogel without a chemical reaction; thus, no new chemical bond could be characterised.

### 3.4. Thermal Analysis

The thermal properties of the hydrogel samples were analysed via DSC (Figure 3c). Similar curves began at ~100 °C and peaked at ~115 °C in all the groups. This peak can be ascribed to the loss of water and the existence of hydrated chitosan. This result was consistent with the results reported by Thanyacharoen et al. [25], and it was described as a glass transition temperature.

### 3.5. Rheological Analysis

The linear visco-elastic range of different concentrations of the β-TCP-loaded chitosan hydrogel showed a similar linear visco-elastic range, where the strain value was between 0.01% and 1%. The strain value at 0.1% was chosen as a parameter to perform the following rheological test.

**Figure 4:**
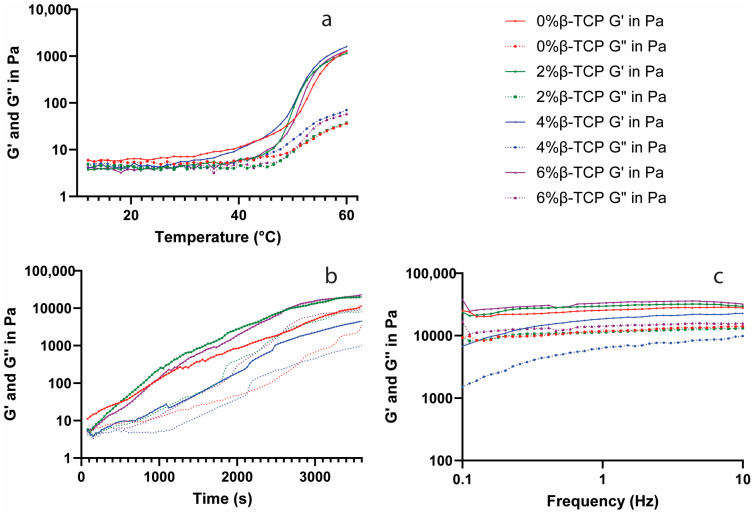
The viscoelastic moduli, G′ and G″, of the tested hydrogel samples as functions of temperature (**a**), time (**b**), and frequency (**c**) are tested separately.

The storage modulus (G′) and loss modulus (G″) as functions of temperature for the β-TCP-loaded chitosan hydrogels were defined (Figure 4a). The G′ and G″ increased rapidly at around 40–45 °C, indicating the temperature-induced gelification process. After increasing for around 3000 s, the G′ and G″ became stable as the functions of time at 37 °C (Figure 4b). The chitosan hydrogels with 2% and 6% β-TCP showed a higher G′ and G″ value compared to the control group, while the 4% β-TCP chitosan hydrogel had the lowest G′ and G″ values. The G′ and G″ were represented independent from the frequency value, and the 4% β-TCP group revealed a lower G′ and G″ when compared with the other groups (Figure 4c).

### 3.6. Degradation Test

**Figure 5:**
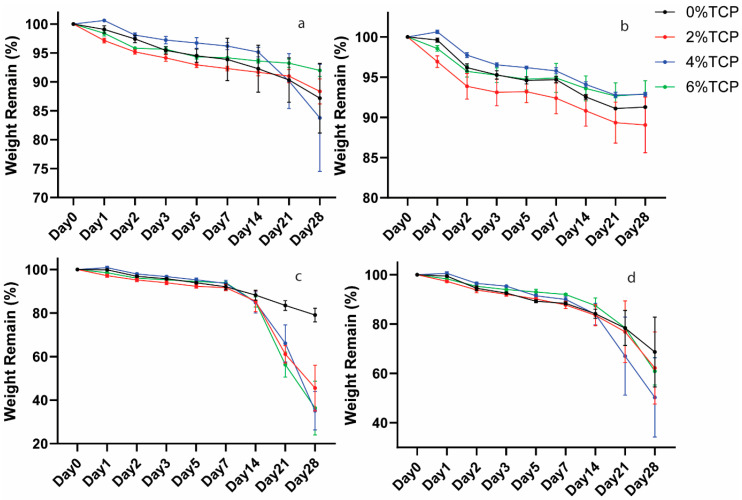
Weight remaining ratios of β-TCP-loaded chitosan hydrogel samples in PBS, AS, 10 μg/mL lysozyme, and 100 μg/mL lysozyme were tested at 37 °C (**a**–**d**) separately.

The degradation behaviour of the test hydrogels in four different media was evaluated by monitoring the remaining weight of the hydrogels as a function of time (Figure 5). All the samples showed a decrease in weight with time, demonstrating the degradability of the hydrogels. The hydrogels retained over 80% of their weight after 4 weeks in the PBS and AS groups and showed a higher weight loss in the lysozyme groups. Incorporation with β-TCP did not affect the degradation ability of the chitosan hydrogels in the PBS, AS, and higher lysozyme concentration groups (Figure 5a,b,d), but had a significantly higher weight loss in the lower lysozyme concentration group (Figure 5c).

### 3.7. In Vitro Studies

**Figure 6:**
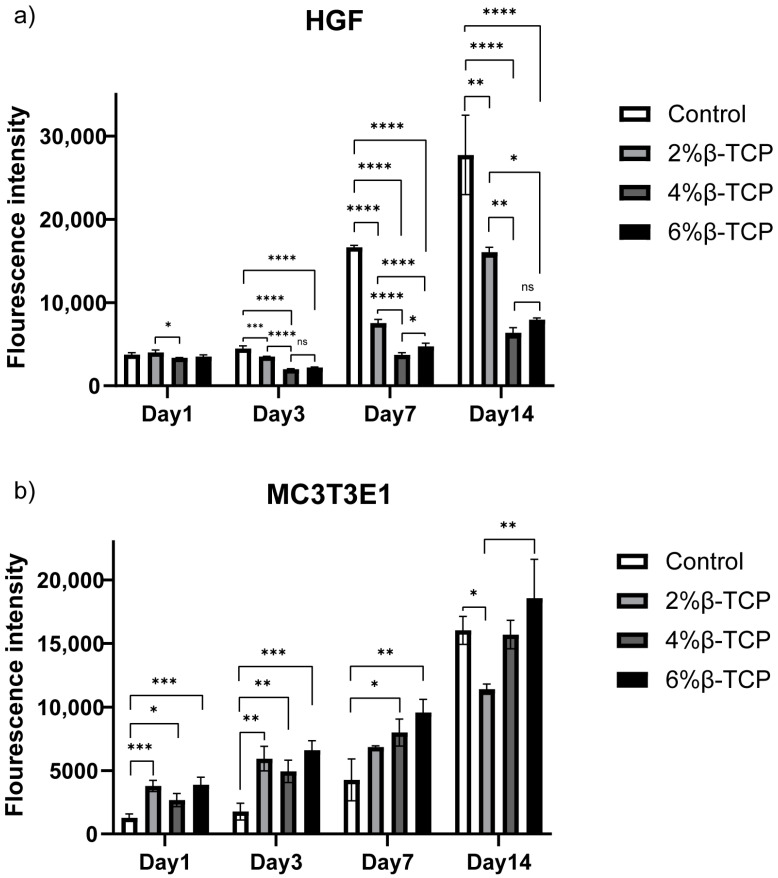
Alamar Blue assay on HGF (**a**) and MC3T3-E1 cells (**b**), 3D cultured in the hydrogel samples. The control group is the hydrogel without β-TCP.

The Alamar Blue analysis results showed the good cell viability of the β-TCP-loaded chitosan hydrogels (Figure 6). Both the MC3T3-E1 cell line and HGF cells proliferated after culturing for 14 days. The hydrogels with β-TCP presented a higher cell viability when culturing MC3T3-E1 cells than non-β-TCP hydrogels in the first three days, and all four groups showed good cell proliferation at day 14. The hydrogels with different concentrations of β-TCP showed similar cell viability behaviour when culturing HGF cells on day 1, whilst the lower concentration of β-TCP-loaded hydrogels exhibited better cell viability compared to the rest. The HGF cells proliferated on day 7 and day 14, with a significantly higher proliferation in the lower concentration of β-TCP-loaded hydrogel groups.

**Figure 7:**
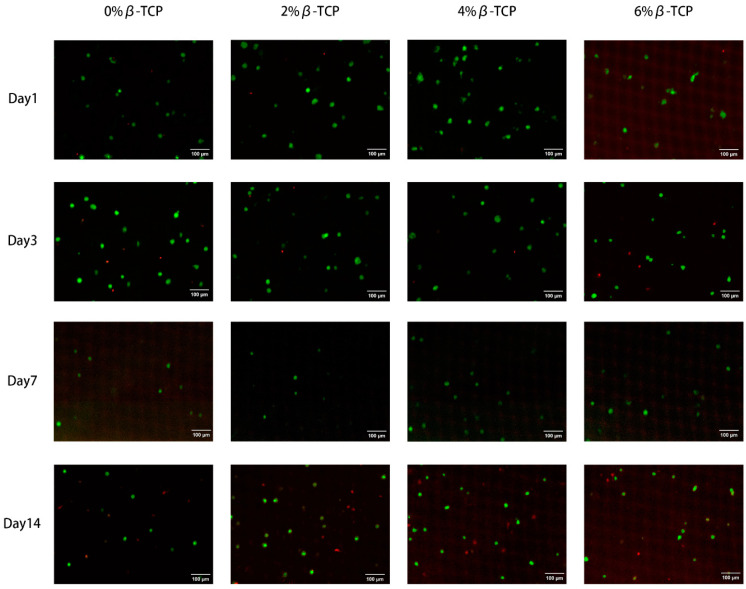
Confocal images of live cells (green) and dead cells (red) show the viability of HGF cells encapsulated in the hydrogel samples after 1, 3, 7, and 14 days in culture. Scale bars: 100 μm.

The confocal images of the HGF cells stained using the Live/Dead kit confirmed the excellent cell viability of the β-TCP-loaded chitosan hydrogels (Figure 7). Most of the cells were viable after culturing for 14 days, as indicated by green fluorescence. The appearance of cells spreading in the green fluorescence showed that the cells were healthy and able to grow.

## 4. Discussion

In this study, β-TCP-loaded chitosan hydrogels were synthesised successfully, as described before [16,26], and their thermosensitive property was confirmed. β-TCP was added successfully into chitosan hydrogels to enhance their mechanical and biological properties.

As a natural polymer, chitosan showed potential in biomaterial and tissue engineering (BTE) applications due to its excellent characteristics and properties [27]. Chitosan solution is able to achieve thermal gelation by adding β-GP to the system. Chitosan is soluble in a weak acid environment due to the amino group from the chitosan, which easily reacts with the acid to form salt, and the neutralisation of the chitosan solution results in the formation of a hydrated gel-like precipitate. The addition of β-GP to the chitosan solution can inhibit the precipitation of the chitosan solution whilst increasing the pH level to a physiological range (7.0 to 7.4). At a low temperature, the aggregation of chitosan chains is blocked by chitosan–water interactions. The water molecules are removed by the glycerol as the temperature increases, which promotes the association of chitosan chains and leads to gel formation [26,28]. Apart from giving the thermo-sensitive ability to the hydrogels, β-GP is also able to promote the differentiation of MSCs into osteoblasts [29]. Increasing the concentration of β-GP can provide a shorter gelation time, but can also cause cytotoxicity [30]. An appropriate concentration of β-GP should be selected to balance the physical and chemical properties and the biological properties of the hydrogels. In our study, 10% (*w*/*v*) β-GP in the chitosan hydrogels demonstrated favourable properties and safety for cells. The temperature-induced phase transition nature overcomes the limitations of preformed scaffolds and broadens the clinical application prospect of the hydrogels. At room temperature, the hydrogels keep at a liquid state and are able to be applied in periodontal pockets and osseous defects, adapting to their configuration. After being injected, and with the environmental temperature increasing to body temperature, the sol–gel transition occurs, and the hydrogel can provide structure for cell proliferation or drug release.

The weak mechanical properties of chitosan, however, limit its application in BTE. Blending with one or more functional materials (polymers/ceramic particles) could overcome this disadvantage and improve the properties of chitosan hydrogels [31]. Calcium phosphates as inorganic materials can provide cell-binding sites, helping cells to adhere to their surface and release ionic species, which can promote cell proliferation, differentiation, and extracellular matrix mineralization [32]. In our study, β-TCP was added into the chitosan hydrogel instead of hydroxyapatite because (1) β-TCP resorbs faster than hydroxyapatite and it can be gradually resorbed by the body, whilst hydroxyapatite remains in the body for a longer period [33,34], and hence, β-TCP has advantages in some applications such as drug delivery compared to hydroxyapatite; (2) β-TCP is often used where rapid bone regeneration is required, but hydroxyapatite is used when long-term structural support is needed [35]. The successful β-TCP/chitosan mixture hydrogels were confirmed with the XRD and FTIR results, and the presence of β-TCP did not inhibit the gelation progress. Our SEM and EDX results also demonstrated that the chitosan hydrogels with β-TCP contained thick fibres connecting the sheets, which can enhance the structure of the hydrogels, and a similar G′ and G″ in the hydrogels with β-TCP compared with the control group, except for the 4% β-TCP group.

Our rheology results showed a higher G′ in the β-TCP-loaded chitosan hydrogels compared with the control group. The G′ and G″ of all four groups showed a rapid increase at around 40–45 °C, which might be due to a short delay (1–2 min) of the gel formation when the temperature reaches 37 °C. The physical crosslinks between chitosan and β-GP give the hydrogels a high degree of elasticity, and the association between the phosphate ions from β-TCP and the amine from chitosan might be the reason why β-TCP-loaded chitosan hydrogels show a higher elastic modulus [36].

Chitosan hydrogels are mainly degraded into amino groups through lysozyme-mediated hydrolysis in vivo [37,38], whilst the degraded monomeric units demonstrated no toxicity and cycling through metabolism [37]. Our degradation test of the chitosan hydrogels was performed in four different media, and all the groups showed considerable resistance to degradation within one month. Incorporation with β-TCP did not give the chitosan hydrogels a better resistance to degradation.

The excellent biocompatibility of the hydrogels was confirmed on two types of cells (HGF and MC3T3-E1), supporting the potential clinical application of the hydrogels in periodontal regenerative therapy. This result is consistent with other in vitro studies [39]. Chitosan hydrogels have already been tested as scaffolds to carry drugs and signalling molecules for the control of inflammation and promoting periodontal regeneration in vivo [11,40]. With the ability to control the load and release of different therapeutic agents, chitosan hydrogels can be regarded as carriers or scaffolds for disease therapy and tissue repair and regeneration [17].

Besides their primary therapeutic purposes, hydrogels are also designed to approach proactive functions, including biomonitoring [41,42], self-healing [43], and cell encapsulating [12,44,45]. The oral and periodontal bacterial community holds promise as a future real-time monitoring target of proactive hydrogels, as changes in the bacterial community in the oral and periodontal environment are strongly associated with the occurrence and progress of periodontal diseases. The ability to provide a multifunction of hydrogels has great advantages to overcome the current treatment, with a singular function, and has vast perspectives in preventing and treating periodontal diseases.

## 5. Conclusions

In conclusion, a novel β-TCP-loaded thermosensitive chitosan hydrogel was successfully synthesised. This hydrogel showed a thermosensitive ability and excellent physical, chemical, and biological properties. A combination of inorganic components mixed with the chitosan hydrogel can strengthen the structure and properties of the hydrogel effectively. These findings suggest that β-TCP-loaded thermosensitive chitosan hydrogels have great potential as a scaffold to prevent or treat periodontal diseases.

## Data Availability

The data that support the findings of this study are available from the corresponding author upon request.
